# Influence of UV Radiation on the Color Change of the Surface of Steamed Maple Wood with Saturated Water Steam

**DOI:** 10.3390/polym14010217

**Published:** 2022-01-05

**Authors:** Ladislav Dzurenda, Michal Dudiak, Eva Výbohová

**Affiliations:** Faculty of Wood Sciences and Technology, Technical University in Zvolen, T.G. Masaryka 24, 96001 Zvolen, Slovakia; xdudiak@tuzvo.sk (M.D.); vybohova@tuzvo.sk (E.V.)

**Keywords:** maple wood, color difference, ATR-FTIR spectroscopy, steaming, saturated water steam

## Abstract

The wood of maple (*Acer Pseudopatanus* L.) was steamed with a saturated steam-air mixture at a temperature of *t* = 95 °C or saturated steam at *t* = 115 °C and *t* = 135 °C, in order to give a pale pink-brown, pale brown, and brown-red color. Subsequently, samples of unsteamed and steamed maple wood were irradiated with a UV lamp in a Xenotest Q-SUN Xe-3-H after drying, in order to test the color stability of steamed maple wood. The color change of the wood surface was evaluated by means of measured values on the coordinates of the color space CIE L* a* b*. The results show that the surface of unsteamed maple wood changes color markedly under the influence of UV radiation than the surface of steamed maple wood. The greater the darkening and browning color of the maple wood by steaming, the smaller the changes in the values at the coordinates L*, a*, b* of the steamed maple wood caused by UV radiation. The positive effect of steaming on UV resistance is evidenced by the decrease in the overall color difference ∆E*. While the value of the total color diffusion of unsteamed maple wood induced by UV radiation is ∆E* = 18.5, for maple wood steamed with a saturated steam-air mixture at temperature *t* = 95 °C the ∆E* decreases to 12.6, for steamed maple wood with saturated water steam with temperature *t* = 115 °C the ∆E* decreases to 10.4, and for saturated water steam with temperature *t* = 135 °C the ∆E* decreases to 7.2. Differential ATR-FTIR spectra declare the effect of UV radiation on unsteamed and steamed maple wood and confirm the higher color stability of steamed maple wood.

## 1. Introduction

The color of wood is a basic physical-optical property, which belongs to the group of macroscopic features on the basis of which the wood of individual woody plants differs visually. The color of the wood is formed by chromophores, i.e., functional groups of the type: >C=O, –CH=CH–CH=CH–, –CH=CH–, aromatic nuclei found in the chemical components of wood (lignin and extractive substances, such as dyes, tannins, resins, etc.), which absorb some components of the electromagnetic radiation of daylight and thus create the color of the wood surface perceived by human vision.

The color of wood changes in thermal processes, such as wood drying, wood steaming, and thermo-wood production technologies. The wood darkens more or less and, depending on the wood, acquires color shades of pink, red, and brown to dark brown-gray color [1,2,3,4,5,6,7,8,9,10,11].

Wood steaming is a physico-chemical process, in which wood placed in an environment of hot water, saturated water steam or saturated humid air is heated and changes its physical, mechanical, and chemical properties. The action of heat initiates the chemical reactions in wet wood, such as the extraction of water-soluble substances, degradation of polysaccharides, cleavage of free radicals, and phenolic hydroxyl groups in lignin, resulting in the formation of new chromophoric groups causing a change in the color of the wood. These facts are used for the full-volume modification of wood color into non-traditional color shades of wood of individual trees. Beech wood, depending on the length of the steaming time, acquires a pale pink to red-brown color shade [5,7,9,12,13,14,15]. Oak wood, as reported by the works [11,16,17] and depending on the steaming conditions, achieves color shades from a pale brown-yellow color to a dark dark-gray color. The light white-yellow color of maple wood in the process of steaming the wood with saturated water steam acquires shades of pale pink-brown to brown-red color [18,19].

The color of the wood also changes due to the long-term effects of sunlight on its surface. The surface of the wood darkens and is mostly yellow and brown. This fact is also referred to in the professional literature as natural aging [20,21,22].

Solar radiation falling on the wood surface is partly absorbed and partly reflected from the surface. The absorbed spectrum of infrared electromagnetic radiation is converted into heat. In addition, the photon flux of ultraviolet and part of the visible radiation of wavelengths *λ* = 200–400 nm are the source of initiation of photolytic and photooxidation reactions with lignin, polysaccharides, and accessory substances of wood. Of the chemical components of wood, lignin is the most subject to photodegradation, which captures 80–85% of UV radiation, while carbohydrates absorb 13–18% and 2% of accessory substances [23]. These reactions cleave the lignin macromolecule with the simultaneous formation of phenolic hydroperoxides, free radicals, carbonyl and carboxyl groups, and to a lesser extent depolymerize polysaccharides to polysaccharides with a lower degree of polymerization to form carbonyl, carboxyl groups, and gaseous products (CO, CO_2_, H_2_). Although the photodegradation of natural wood is a widely studied phenomenon [20,22,24,25,26,27,28,29], less attention has been paid to the issue of photodegradation and color stability of steamed wood.

The aim of the work is to investigate the color fastness of maple wood obtained by the process of steaming with a saturated steam-air mixture or with saturated water steam through a simulated aging process-UV radiation in Xenotest Q-SUN Xe-3-H. The color fastness of the wood is evaluated by changes in the coordinates L*, a*, b* of the color space CIE L* a* b*, the total color difference ∆E*, and changes in the values of differential absorbance *A_d_* of selected bands in FTIR spectra.

## 2. Material and Methods

### 2.1. Material

The wet wood of maple blanks with the following dimensions: Thickness: *h* = 40 mm, width *w* = 100 mm, length *d* = 750 mm, and moisture content *w_p_* = 57.8 ± 4.8%, was steamed with a saturated steam-air mixture at a temperature of *t* = 95 °C or saturated steam at *t* = 115 °C and *t* = 135 °C for *τ* = 9 h, in order to obtain a pale pink-brown, pale brown, and brown-red color in a pressure autoclave: APDZ 240 in Sundermann s.r.o. (Banská Štiavnica, Slovakia). The steamed and unsteamed maple wood blanks were subsequently dried to a moisture content of *w* = 10 ± 0.5%. Samples measures with the following dimensions: Thickness: *h* = 15 mm, width *w* = 50 mm, and length *d* = 100 mm, were made to test the color fastness of the wood.

### 2.2. Color Measurement of Maple Wood

The color of steamed and unsteamed maple wood before and after irradiation was measured in the color space CIE L*a*b*. To measure the color of maple wood, in the color space CIE L*a*b*, the color reader CR-10 (Konica Minolta, Osaka, Japan) was measured. A D65 light source was used and the diameter of the optical scanning aperture was 8 mm.

The color measurement was performed on a radial surface machined by planning. The color coordinates of maple wood samples in the color space CIE L*a*b* before irradiation are given in Table 1.

The total color difference ΔE* of the color change of the surface of the maple wood samples under the influence of UV radiation is determined according to the following equation ISO 11 664-4:

(1) $$\Delta E^{*}=\sqrt{{\left(L_{298}^{*}-L_{0}^{*}\right)}^{2}+{\left(a_{298}^{*}-a_{0}^{*}\right)}^{2}+{\left(b_{298}^{*}-b_{0}^{*}\right)}^{2}}$$

where L^*^_0_, a^*^_0_, b^*^_0_ are values on the coordinates of the color space of the surface of the dried milled native and thermally treated maple wood before exposure.

L^*^_298_, a^*^_298_, b^*^_298_ are values on the surface color coordinates of the dried milled native and thermal treated maple wood during UV exposure.

### 2.3. Irradiation of Maple Wood in Xenon Test Chamber

In the Q-SUN Xe-3-H Xenon test chamber, Q-Lab Corporation, Westlake, OH, USA (1800 W Xenon arc lamp-full spectrum, irradiation 0.35 W/m^2^-340 nm, black panel temperature 63 °C), the samples were irradiated for *τ* = 298 h. During the exposure, the color of the irradiated surface was measured regularly at *τ* = 24 h intervals.

### 2.4. Analysis of Changes in Lignin-Cellulose Matrix of Wood ATR-FTIR Spectroscopy

Infrared spectroscopy was used to monitor changes in maple wood components induced by UV radiation in unsteamed and steamed wood. The FTIR surface analysis of wood samples was performed on a Nicolet iS 10 FTIR spectrometer (Thermo Fisher Scientific, Madison, WI, USA) using the attenuated total reflectance (ATR-FTIR) technique. The measurements were performed on a diamond crystal in the range of 4000–650 cm^−1^. For each sample, 64 scans were performed at a resolution of 4 cm^−1^. The obtained spectral records were evaluated by the spectroscopic software OMNIC 8. The calculation of the values of the differential absorbance *A_d_* describes the relation:

(2) $$A_{d}=\frac{\left(A_{i}-A_{ref}\right)}{A_{ref}}\cdot 100$$

where *A_d_* is the differential absorbance, *A_i_* is the absorbance at a given wavelength in the spectrum of the irradiated sample, and *A_ref_* is the absorbance at a given wavelength in the spectrum of the unirradiated sample.

## 3. Results and Discussion

### 3.1. Color Analysis

**Figure 1:**
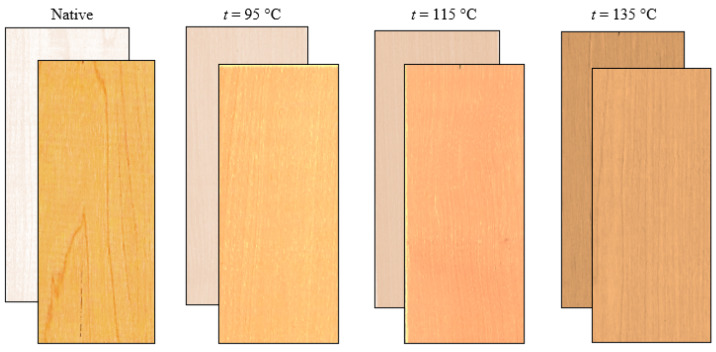
View of maple wood before and after UV irradiation: Native; steamed at *t* = 95 °C; steamed at *t* = 115 °C; steamed at *t* = 135 °C.

The color of unsteamed and steamed maple wood before and after UV irradiation in the Q-SUN Xe-3-H test chamber (Q-Lab Corporation, Westlake, OH, USA) is shown in Figure 1.

According to the visual evaluation of the color of maple wood before and after the UV radiation, it can be stated that while the light white-yellow color of untreated maple wood darkens and acquires a yellow-red-brown color shade due to photodegradation reactions induced by UV radiation, which is the pale pink-brown of court wood treated with a steam-air mixture with a temperature of *t* = 95 °C, it darkened slightly with UV radiation and took on a pale brown-yellow color shade. The brown-red color of steamed maple wood obtained by steaming with saturated steam with a temperature of *t* = 135 °C lightened.

**Figure 2:**
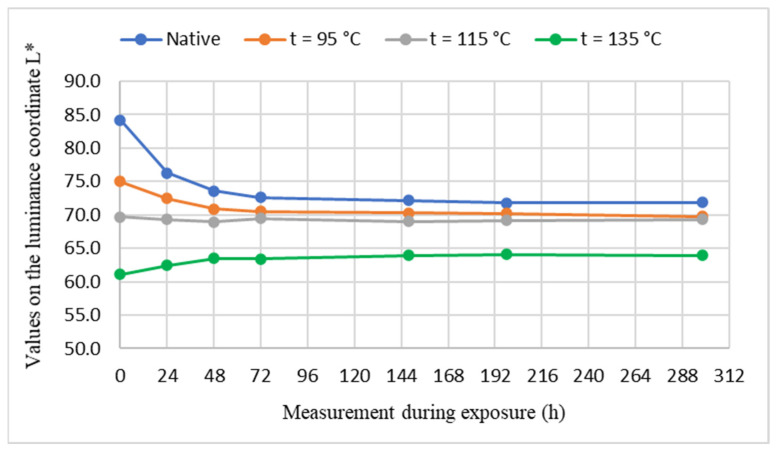
The course of changes of values on the light coordinate L* in the process of UV irradiation of samples of unsteamed and steamed maple wood.

**Figure 3:**
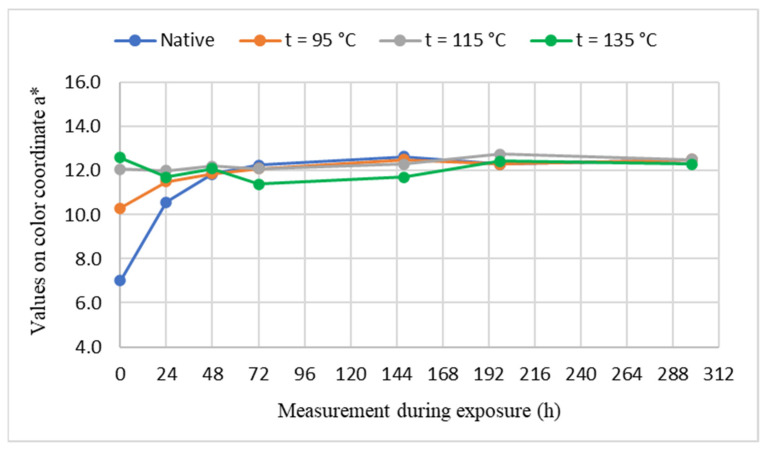
The course of changes of values on the coordinate of red color and a* in the process of UV irradiation of samples of unsteamed and steamed maple wood.

**Figure 4:**
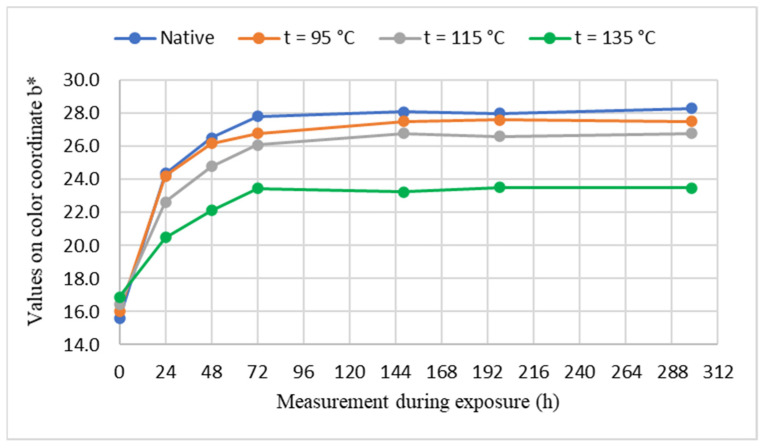
The course of changes of values on the coordinate of yellow color b* in the process of UV irradiation of samples.

The course of color changes of unsteamed and steamed maple wood in the color space CIE L*a*b* under the influence of UV radiation in Xenotest Q-SUN Xe-3-H for 298 h, are shown in Figure 2, Figure 3 and Figure 4.

Based on the experimentally determined values of color changes on the luminance coordinate L*, which are the chromaticity coordinates red a* and yellow color b* of maple wood samples induced by photodegradation reactions of individual maple wood components with UV radiation in Xenotest Q-SUN Xe-3-H, it can be stated that significant changes in the color of the wood occur in the first 72 h of UV radiation. The greater the darkening and browning color of the maple wood by steaming, the smaller the changes in the values at the coordinates L*, a*, b* of the steamed maple wood caused by UV radiation. The greater the darkening and browning color of the maple wood by steaming, the smaller the changes in the values at the coordinates L*, a*, b* of the steamed maple wood caused by UV radiation. The change of color of unsteamed maple wood is greater than the steamed maple wood. Numerically, this is documented by the shifts on the individual coordinates of the color space CIE L*a*b* of the analyzed maple wood samples before and after UV irradiation, as shown in Table 2.

The degree of darkening and browning of the unmapped maple during 298 h of UV irradiation in the CIE L*a*b* color space is declared by a decrease in the luminance coordinate by ΔL* = −12.3 and an increase in points in the red chromatic coordinate by ∆a* = +5.8 a on the yellow coordinate b* by the value ∆b* = +12.7. The above findings on darkening of wood due to UV radiation are in accordance with the opinions of experts dealing with changes in the properties of wood due to solar radiation, respectively UV radiation is shown in [20,22,24,25,29,30,31].

The differences in the color of steamed maple wood before and after UV irradiation on the light coordinate L* and the chromaticity coordinate of red a* and yellow b* are smaller compared to the changes of unsteamed maple wood.

While the darkness of the thermal treated maple wood with the steam-air mixture with the temperature *t* = 95 °C due to UV radiation increased, the darkness of the maple wood treated with the saturated steam increased by decreasing the values from *L*_0_^*^ = 75 to *L*_298_^*^ = 69.8, i.e., ∆L* = −5.2 temperature *t* = 115 °C did not change due to UV radiation and the brightness of steamed maple wood with saturated water steam with temperature *t* = 135 °C increased from *L*_0_^*^ = 61.1 to *L*_298_^*^ = 63.9, i.e., the value of ∆L* = +2.8. The decrease in the darkening of maple wood steamed at higher steaming temperatures due to UV radiation or to achieving the opposite effect—lightening the surface of steamed maple wood with saturated steam with temperature *t* = 135 °C indicates changes in the chromatic system caused by steaming, which affects the photochemical reactions of UV radiation with functional groups of the chromophore system of steamed maple wood. The work of [32,33] also points that steamed wood, unlike unsteamed wood, is more or less resistant to UV radiation.

The effect of fading of the red-brown color of the beech wood surface is achieved by steaming with a saturated steam with temperature *t* = 120 ± 2 °C after UV irradiation in the Xenotest 450 Xenon lamp. This emits UV radiation with a wavelength of 340 nm and an intensity of 42 ± 2 W/m^2^ for 7 days, as stated in the work of [32].

In [33], the effect of UV radiation on steamed agate wood states that while the surface of steamed agate wood darkened slightly at a steaming temperature *t* = 100 °C, the surface of agate wood brightened at a steaming temperature *t* = 120 °C.

The positive effect of maple wood steaming on the resistance to the effects of UV radiation is declared by the decrease in values ∆a* and ∆b* on the chromatic coordinates given in Table 2. The pale pink-brown color obtained by steaming with a steam-air mixture with temperature *t* = 95 °C by absorbing UV radiation increased on the red coordinate by a* = +2.1. In addition, the pale-brown color of steamed maple wood formed by steaming with saturated steam with temperature *t* = 115 °C due to UV radiation increased by the value ∆a* = +0.6. Moreover, the effect of UV radiation on the surface of steamed maple wood steamed with saturated steam with a temperature of *t* = 135 °C was manifested by a decrease in the value on the red coordinate from *a*_0_^*^ = 12.6 to *a*_298_^*^ = 12.3, i.e., value ∆a* = −0.3. Similarly, at the yellow coordinate there are decreases in ∆b* values caused by UV radiation on steamed maple wood. The value of the change ∆b* on the yellow coordinate induced by UV radiation on the surface of maple wood steamed with a steam mixture with temperature *t* = 95 °C is ∆b* = +11.5. In addition, maple wood steamed with saturated water steam with temperature *t* = 115 °C is ∆b* = +10.4 and saturated water steam with temperature *t* = 135 °C is ∆b* = +6.6. Based on the above findings, it can be stated that the functional groups of the maple wood chromophoric system absorbing electromagnetic radiation spectra with a wavelength of red 630–750 nm and a wavelength of 570–590 nm of yellow color were significantly eliminated for photochemical reactions of wood induced by UV radiation for the red color and to a lesser extent for the yellow color.

**Figure 5:**
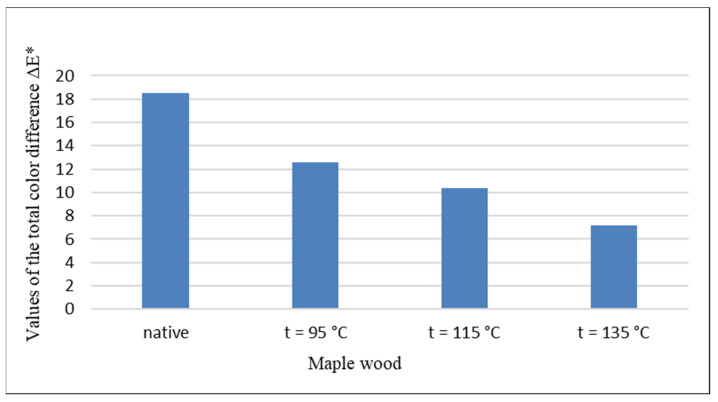
Influence of UV radiation on the size of the total color difference ∆E* of unsteamed and steamed maple wood.

The changes in the values at the individual coordinates of the color space CIE L*a*b* induced on the surface of unsteamed and steamed maple wood by UV radiation in Xenotest 450 are reflected in the quantification of the color change of the maple wood surface expressed by the total color difference ∆E*. The influence of UV radiation on the magnitude of color changes of the analyzed maple wood samples in the form of the total color difference ∆E* is shown in Figure 5.

The lower values of the total color difference ∆E* of steamed maple wood indicate the benefit of steaming on the resistance of steamed maple wood to UV radiation causing the color change in the process of natural aging. While the color change of unsteamed maple wood caused by UV radiation reaches the value ∆E* = 18.5, for steamed maple wood steamed with temperature *t* = 95 °C it is ∆E* = 12.6, which is a decrease of 31.8% compared to the total color difference of unsteamed maple wood, for steamed maple wood with saturated steam with temperature *t* = 115 °C is ∆E* = 10.4, which is a decrease of 43.8%, and for steamed maple wood steamed with saturated steam with temperature *t* = 135 °C is ∆E* = 7.2, which is a decrease of 61.1%.

### 3.2. ATR-FTIR Spectroscopy Analysis

**Figure 6:**
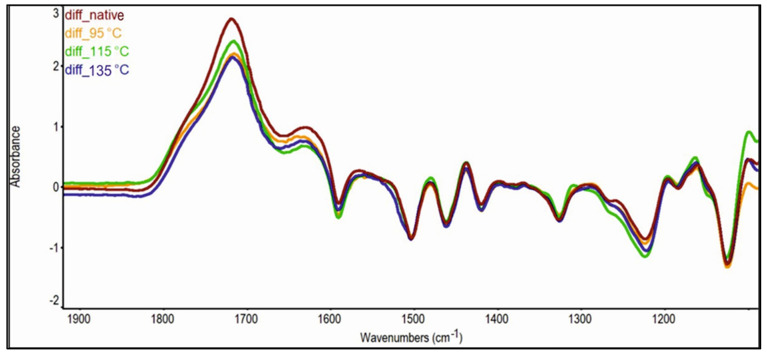
Differential ATR-FTIR spectra expressing the effect of UV radiation on unsteamed and steamed maple wood.

The color changes of maple wood samples caused by photodegradation reactions initiated by UV radiation are also documented by FTIR analyses of the surface of unsteamed and steamed maple wood after UV radiation in Figure 6 and Table 3.

The results of FTIR analysis indicate the formation of new carbonyl C=O groups in the spectra of the samples manifested by an increase in the intensity of the absorption band at a maximum of 1720 cm^−1^. In this section, an overlap of several absorption bands can be observed, which is a manifestation of vibrations of conjugated and unconjugated C=O bonds, as well as carboxyl groups. These can come not only from the main constituents of wood (lignin, cellulose, hemicelluloses), but also from extractives [33]. We recorded the most significant increase in the intensity of the C=O group bands in the spectrum of the irradiated native sample, by more than 57%. In the case of irradiated steamed wood samples, this increase ranges from 16 to 21%.

The performed FTIR analyses also indicate a significant degradation of the lignin macromolecule due to UV radiation. After irradiating the steamed wood sample at 135 °C, we recorded a complete loss of the absorption band at wavenumber 1504 cm^−1^, in order to be able to speak of complete degradation of lignin in the surface layer of the samples. After irradiation of the samples steamed at 95 and 115 °C, the intensity of the said characteristic lignin absorption band decreased by 96.24% or 97.37%. The reduction can also be observed by comparing the intensities of other absorption bands characteristic for lignin, at wavenumber 1593, 1462, 1422, 1225, and 830 cm^−1^. However, it should be noted that at 1462 and 1225 cm^−1^, not only vibrations of lignin, but also hemicelluloses, occur.

Several studies have confirmed that lignin is the most sensitive to UV radiation among all of the wood components [24,29,34,35]. By absorbing energy, the bonds are cleaved and new functional groups (carbonyl and carboxyl) are formed, as well as radicals, which further induce lignin depolymerization and condensation reactions. Aromatic phenoxyl radicals react with oxygen to form unsaturated carbonyl compounds (quinones), which contribute to the color changes of wood [25,36].

Based on the decrease in the intensities of the absorption bands at the wavenumber 1328 and 1126 cm^−1^, we can state that in addition to lignin, polysaccharide degradation also occurs. While the first band corresponds to the vibrations of cellulose macromolecule, the second band belongs to the symmetric valence vibrations of the ether bond and the glucose ring [33,37].

Since the formation of new C=O bonds is considered to be the main cause of wood color changes during its exposure to UV radiation, the results of FTIR analysis confirm the positive effect of thermal steaming of wood on its color stability.

## 4. Conclusions

The surface color of unsteamed maple wood changes more markedly than the surface color of steamed maple wood due to UV radiation. The more pronounced the darkening and browning color of the steamed maple wood, the smaller the UV-induced changes in the color of the steamed maple wood. This is evidenced by the degree of darkening of the surface of unsteamed and steamed maple wood at *t* = 95 °C and *t* = 125 °C after UV irradiation by decreasing values on the luminance coordinate L*, as well as the rate of decrease of ∆a*, ∆b* values on chromatic coordinates. The decrease in ∆a* and ∆b* values on the chromatic coordinates indicates that the functional groups of the maple wood chromophore system absorbing electromagnetic radiation spectra with a wavelength of red of 630–750 nm and a wavelength of 570–590 nm were eliminated to a lesser extent by steaming for photochemical reactions of wood caused by UV radiation.

The positive effect of maple wood steaming on the limiting effect of initiating photo degradation reactions induced by UV radiation on the surface of steamed maple wood is evidenced by the decrease in the overall color difference ∆E*. While the change in color of unsteamed maple wood caused by UV radiation expressed by the total color difference is ∆E* = 18.5, for steamed maple wood the stated changes in color difference values depending on the steaming temperature decrease from ∆E* = 12.6 to ∆E* = 7.2, which is a decrease from 31.8 to 61.1%.

This is confirmed by the results of FTIR analyses. While in the case of unsteamed wood we recorded an increase in the intensity of absorption bands of chromophoric C=O groups due to UV radiation by more than 57%, in the case of steamed wood samples this increase is lower and ranges from 16 to 21%.

## Figures and Tables

**Table 1:** Coordinate values of color space CIE L*a*b* of native and thermally treated maple wood.

Maple Wood	Wood Color	Color Coordinates in the Color Space CIE L*a*b*		
		L*	a*	b*
unsteamed maple wood	pale white-yellow	84.2 ± 2.4	7.1 ± 1.5	15.6 ± 1.9
steamed at *t* = 95 ± 2.5 °C	pale pink-brown	75.0 ± 1.8	10.3 ± 1.4	16.0 ± 1.6
steamed at *t* = 115 ± 2.5 °C	light brown	69.7 ± 1.6	11.9 ± 1.4	16.4 ± 1.4
steamed at *t* = 135 ± 2.5 °C	brown-red	61.1 ± 1.4	12.6 ± 1.3	16.9 ± 1.6

**Table 2:** Sizes of changes in ∆L*, ∆a*, ∆b* values in the CIE L*a*b* color space of unsteamed and steamed maple wood before and after UV irradiation in the Q-SUN Xe-3-H test chamber.

Maple Wood	Color Coordinates of Samples in the CIE L*a*b* Sample Area before and after UV Irradiation in the Q-SUN Xe-3-H Test Chamber								
	*L*_0_^*^	*L*_298_^*^	∆L*	*a*_0_^*^	*a*_298_^*^	∆a*	*b*_0_^*^	*b*_289_^*^	∆b*
unsteamed maple wood	84.2	71.9	−12.3	7.0	12.5	+5.8	15.6	28.3	+12.7
steamed at *t* = 95 ± 2.5 °C	75.0	69.8	−5.2	10.3	12.5	+2.1	16.0	27.5	+11.5
steamed at *t* = 115 ± 2.5 °C	69.7	69.3	−0.4	11.9	12.5	+0.6	16.4	26.8	+10.4
steamed at *t* = 135 ± 2.5 °C	61.1	63.9	+2.8	12.6	12.3	−0.3	16.9	23.5	+6.6

**Table 3:** Values of differential absorbances of selected absorption bands expressing the influence of UV radiation on unsteamed and steamed maple wood.

Maple Wood Samples	*A*_*d* (1720)_ (%)	*A*_*d* (1504)_ (%)	*A*_*d* (1235)_ (%)
unsteamed maple wood	157.23	−96.02	−25.30
steamed at *t* = 95 ± 2.5 °C	116.80	−96.24	−31.56
steamed at *t* = 115 ± 2.5 °C	121.43	−97.37	−35.04
steamed at *t* = 135 ± 2.5 °C	118.23	−100.00	−31.69

## Data Availability

The data presented in this study are available on request from the corresponding author.
